# Supplementary material for: Exercise routine change is associated with prenatal depression scores during the COVID-19 pandemic among pregnant women across the United States
Source: PLoS One. 2020 Dec 21;15(12):e0243188. doi: 10.1371/journal.pone.0243188 (PMC7751871; doi:10.1371/journal.pone.0243188)
Supplement: Attachment — Submitted filename: AAA-PONE-D-20-29604_reviewer-110720.pdf [file pone.0243188.s003.pdf]

PLOS ONE

Exercise routine change is associated with prenatal depression scores during the COVID-19 pandemic among pregnant women across the United States

--Manuscript Draft--

**Manuscript Number:**	PONE-D-20-29604
**Article Type:**	Research Article
**Full Title:**	Exercise routine change is associated with prenatal depression scores during the COVID-19 pandemic among pregnant women across the United States
**Short Title:**	COVID-19-related exercise change and depression by location
**Corresponding Author:**	Theresa E. Gildner<br>Dartmouth College<br>Hanover, NH UNITED STATES
**Keywords:**	Perinatal depression; coronavirus; pregnancy; mental health; physical activity
**Abstract:**	Background<br><br>The COVID-19 pandemic has negatively affected physical and mental health worldwide. Pregnant women already exhibit an elevated risk for depression compared to the general public, a pattern expected to be exacerbated by the pandemic. Certain lifestyle factors, including moderate exercise, may help support mental health during pregnancy (e.g., by improving mood and increasing perceptions of control), but it is unclear how the pandemic may be impacting these associations across different locations. Here, we test whether: (i) altered exercise routines during the pandemic are associated with higher depression scores; and, (ii) women living in metro areas are more likely to report exercise changes compared to those in non-metro areas, potentially due to having limited space (both indoors and outdoors) for safe exercise.<br><br>Methods<br><br>This cross-sectional study uses data from the COVID-19 And Reproductive Effects (CARE) study, an online survey of pregnant women in the United States. Participants were recruited April-June 2020 (n = 1,862). Linear regression analyses assessed whether COVID-19-related exercise change was associated with depression score as measured by the Edinburgh Postnatal Depression Survey. Logistic regression analyses tested whether a participant's Rural-Urban Continuum Code classification of "metro" was linked with higher odds of reporting exercise changes compared to a "non-metro" classification.<br><br>Results<br><br>Women who reported exercise changes during the pandemic exhibited significantly higher depression scores compared to those reporting no changes. Moreover, individuals living in metro areas of all sizes were significantly more likely to report exercise changes compared to women living in non-metro areas.<br><br>Conclusions<br><br>These results suggest moderate exercise may represent a non-pharmaceutical tool for supporting maternal mental health. Providers should explicitly ask patients how the pandemic has impacted their exercise routines and consider this a risk factor for depression. Efforts should be made to recommend exercises that are tailored to individual living conditions and physical health.
**Order of Authors:**	Theresa E. Gildner
	Elise J. Laugier
	Zaneta M. Thayer
**Additional Information:**	

Powered by Editorial Manager® and ProduXion Manager® from Aries Systems Corporation

Question	Response
**Financial Disclosure**<br><br>Enter a financial disclosure statement that describes the sources of funding for the work included in this submission. Review the submission guidelines for detailed requirements. View published research articles from *PLOS ONE* for specific examples.<br><br>This statement is required for submission and **will appear in the published article** if the submission is accepted. Please make sure it is accurate.<br><br>**Unfunded studies**<br>Enter: *The author(s) received no specific funding for this work.*<br><br>**Funded studies**<br>Enter a statement with the following details:<br>• Initials of the authors who received each award<br>• Grant numbers awarded to each author<br>• The full name of each funder<br>• URL of each funder website<br>• Did the sponsors or funders play any role in the study design, data collection and analysis, decision to publish, or preparation of the manuscript?<br>• **NO** - Include this sentence at the end of your statement: *The funders had no role in study design, data collection and analysis, decision to publish, or preparation of the manuscript.*<br>• **YES** - Specify the role(s) played.<br><br>* typeset	Dr. Zaneta Thayer was funded by the Wenner-Gren Hunt Fellowship (grant #9687) during this project. Participant compensation was provided through the Claire Garber Goodman Fund in the Department of Anthropology at Dartmouth College.
**Competing Interests**<br><br>Use the instructions below to enter a competing interest statement for this submission. On behalf of all authors, disclose any competing interests that could be perceived to bias this work—acknowledging all financial support and any other relevant financial or non-financial competing interests.<br><br>This statement **will appear in the published article** if the submission is	The authors have declared that no competing interests exist.

accepted. Please make sure it is accurate. View published research articles from *PLOS ONE* for specific examples.<br><br>**NO authors have competing interests**<br><br>Enter: *The authors have declared that no competing interests exist.*<br><br>**Authors with competing interests**<br><br>Enter competing interest details beginning with this statement:<br><br>*I have read the journal's policy and the authors of this manuscript have the following competing interests: [insert competing interests here]*<br><br>* typeset	
**Ethics Statement**<br><br>Enter an ethics statement for this submission. This statement is required if the study involved:<br><br>• Human participants<br>• Human specimens or tissue<br>• Vertebrate animals or cephalopods<br>• Vertebrate embryos or tissues<br>• Field research<br><br>Write "N/A" if the submission does not require an ethics statement.<br><br>General guidance is provided below. Consult the submission guidelines for detailed instructions. **Make sure that all information entered here is included in the Methods section of the manuscript.**	This study received ethical approval from Dartmouth College (STUDY00032045). Study information (e.g., study summary, purpose, benefits, risks, and privacy protection details) was provided to all participants prior their completion of the survey. Informed consent was collected by asking potential participants to click a box saying that they had read the study information and consented to participating in the study.

Powered by Editorial Manager® and ProduXion Manager® from Aries Systems Corporation

**Format for specific study types**<br><br>**Human Subject Research (involving human participants and/or tissue)**<br>• Give the name of the institutional review board or ethics committee that approved the study<br>• Include the approval number and/or a statement indicating approval of this research<br>• Indicate the form of consent obtained (written/oral) or the reason that consent was not obtained (e.g. the data were analyzed anonymously)<br><br>**Animal Research (involving vertebrate animals, embryos or tissues)**<br>• Provide the name of the Institutional Animal Care and Use Committee (IACUC) or other relevant ethics board that reviewed the study protocol, and indicate whether they approved this research or granted a formal waiver of ethical approval<br>• Include an approval number if one was obtained<br>• If the study involved *non-human primates*, add *additional details* about animal welfare and steps taken to ameliorate suffering<br>• If anesthesia, euthanasia, or any kind of animal sacrifice is part of the study, include briefly which substances and/or methods were applied<br><br>**Field Research**<br><br>Include the following details if this study involves the collection of plant, animal, or other materials from a natural setting:<br>• Field permit number<br>• Name of the institution or relevant body that granted permission	
**Data Availability**<br><br>Authors are required to make all data underlying the findings described fully available, without restriction, and from the time of publication. PLOS allows rare exceptions to address legal and ethical concerns. See the PLOS Data Policy and FAQ for detailed information.	No - some restrictions will apply

A Data Availability Statement describing where the data can be found is required at submission. Your answers to this question constitute the Data Availability Statement and **will be published in the article**, if accepted.<br><br>**Important:** Stating 'data available on request from the author' is not sufficient. If your data are only available upon request, select 'No' for the first question and explain your exceptional situation in the text box.<br><br>Do the authors confirm that all data underlying the findings described in their manuscript are fully available without restriction?	
**Describe where the data may be found in full sentences. If you are copying our sample text, replace any instances of XXX with the appropriate details.**<br><br>• If the data are **held or will be held in a public repository**, include URLs, accession numbers or DOIs. If this information will only be available after acceptance, indicate this by ticking the box below. For example: *All XXX files are available from the XXX database (accession number(s) XXX, XXX.).*<br>• If the data are all contained **within the manuscript and/or Supporting Information files**, enter the following: *All relevant data are within the manuscript and its Supporting Information files.*<br>• If neither of these applies but you are able to provide **details of access elsewhere**, with or without limitations, please do so. For example:<br><br>*Data cannot be shared publicly because of [XXX]. Data are available from the XXX Institutional Data Access / Ethics Committee (contact via XXX) for researchers who meet the criteria for access to confidential data.*<br><br>*The data underlying the results presented in the study are available from (include the name of the third party*	CARE Study Data Sharing Policy<br><br>The CARE study dataset contains sensitive and potentially identifying participant information. Ethical and legal restrictions therefore prevent us from posting our data to a third-party server. However, data and statistical code will be made available to qualified researchers upon reasonable request. Researchers must agree to privacy and data use expectations that conform to IRB requirements and are central to the welfare of study participants. Requests for data can be easily made using a request form on the study website (https://sites.dartmouth.edu/care2020/request-data-access/).

and contact information or URL).<br>• This text is appropriate if the data are owned by a third party and authors do not have permission to share the data.<br><br>* typeset	
Additional data availability information:	

Manuscript

Click here to access/download;Manuscript;Gildner et al. Exercise and depression by location.docx

Exercise routine change is associated with prenatal depression scores during the COVID-19 pandemic among pregnant women across the United States

Running title: COVID-19-related exercise change and depression by location

Keywords: Perinatal depression; coronavirus; pregnancy; mental health; physical activity

Theresa E. Gildner*[1,2], Elise J. Laugier[1,3], Zaneta M. Thayer[1,3]

1. Department of Anthropology, Dartmouth College, Hanover, NH, United States
2. Department of Anthropology, Washington University in St. Louis, St. Louis, MO, United States
3. Ecology, Evolution, Environment & Society Program, Dartmouth College, Hanover, NH, United States

*Corresponding author

Email: Theresa.E.Gildner@dartmouth.edu (TEG)

Abstract

Background: The COVID-19 pandemic has negatively affected physical and mental health worldwide. Pregnant women already exhibit an elevated risk for depression compared to the general public, a pattern expected to be exacerbated by the pandemic. Certain lifestyle factors, including moderate exercise, may help support mental health during pregnancy (e.g., by improving mood and increasing perceptions of control), but it is unclear how the pandemic may be impacting these associations across different locations. Here, we test whether: (i) altered exercise routines during the pandemic are associated with higher depression scores; and, (ii) women living in metro areas are more likely to report exercise changes compared to those in non-metro areas, potentially due to having limited space (both indoors and outdoors) for safe exercise.

Methods: This cross-sectional study uses data from the COVID-19 And Reproductive Effects (CARE) study, an online survey of pregnant women in the United States. Participants were recruited April-June 2020 (n = 1,862). Linear regression analyses assessed whether COVID-19-related exercise change was associated with depression score as measured by the Edinburgh Postnatal Depression Survey. Logistic regression analyses tested whether a participant's Rural-Urban Continuum Code classification of "metro" was linked with higher odds of reporting exercise changes compared to a "non-metro" classification.

Results: Women who reported exercise changes during the pandemic exhibited significantly higher depression scores compared to those reporting no changes. Moreover, individuals living in metro areas of all sizes were significantly more likely to report exercise changes compared to women living in non-metro areas.

Conclusions: These results suggest moderate exercise may represent a non-pharmaceutical tool for supporting maternal mental health. Providers should explicitly ask patients how the pandemic has

impacted their exercise routines and consider this a risk factor for depression. Efforts should be made to recommend exercises that are tailored to individual living conditions and physical health. (300/300 words)

Author summary

The COVID-19 pandemic has negatively impacted the well-being of people across the U.S. and has been linked with elevated depression rates. However, certain groups may be disproportionally impacted, including pregnant women. Given the relatively high rates of depressive symptomology evident among pregnant women prior to the pandemic, supporting the maternal mental health during these challenging times is especially important. However, fetal exposure to psychotropic medications taken by the mother limits their utility, necessitating the use of non-pharmaceutical treatments, such as moderate exercise, to improve mental health outcomes. Yet, regular exercise routines have likely been affected by the pandemic – especially for those living in densely-populated areas where space to exercise safely is limited – but these associations are not well tested among pregnant women. Here, using data from an online survey of 1,862 women living in the U.S., we find that women who reported exercise changes during the pandemic exhibited higher depression scores compared to those able to maintain their exercise routine. Moreover, individuals living in metro areas were more likely to report exercise changes compared to women living in non-metro areas. These findings suggest an association exists between ability to maintain an exercise routine during the pandemic and maternal depression risk. (200/200 words)

Introduction

The COVID-19 pandemic has impacted the health and livelihoods of people around the world. However, certain countries have been hit harder than others, with the United States exhibiting the highest prevalence and mortality estimates globally. As of September 19, 2020, 6,749,092 people living in the U.S. are estimated to have been infected with the novel coronavirus SARS-CoV-2, while 198,940 are thought to have died from the resulting disease COVID-19 (1). Within the U.S., certain populations appear to be at higher risk for negative COVID-19 disease outcomes, including pregnant women. Although the specific effects of COVID-19 on pregnancy are still being tested, some researchers argue pregnant women are generally at higher risk for viral respiratory illness (2). Moreover, other studies indicate that COVID-19 may be associated with an elevated risk for placental injury, pre-eclampsia, preterm birth, and low birth weight (3–5). These recent findings led the CDC to add pregnancy as a risk factor for severe COVID-19 outcomes on June 25, 2020 (6).

In addition to potentially being at an elevated risk for poor COVID-19 health outcomes, the CDC cautions that pregnant women may feel increased stress or anxiety during the pandemic (6). The physical distancing and “stay at home” orders implemented by many local governments to slow the spread of SARS-CoV-2 are thought to increase feelings of anxiety and isolation, especially among already vulnerable populations (7). Pregnancy-associated physiological changes have been clearly linked with increased depression risk, such that 1 in 8 women in the U.S. have been estimated to experience postpartum depression symptoms (8). These depression-related symptoms include fatigue, changes to appetite or sleep, crying more often than usual, withdrawing from loved ones or the baby, feelings of anger, sorrow, hopelessness, worthlessness, or restlessness, and suicidal ideology (8).

Recent evidence suggests that maternal depression symptomatology has become even more common during the COVID-19 pandemic. Specifically, one study comparing mental health among

pregnant women/new mothers before and after the onset of the pandemic found that rates of clinical depression and moderate to high anxiety significantly increased with the onset of the pandemic (7). These changes appear to be directly linked with the disruptions to daily routines, social isolation, and fears of developing COVID-19, highlighting the need for increased maternal health screening and treatment (7). Other factors, including increased COVID-19-related financial stress or changes to working plans in pregnancy have also been associated with increased depression (9,10). Given the probable exposure of offspring to any psychotropic medications taken by the mother during pregnancy or breastfeeding (11), the identification of non-pharmaceutical treatments to reduce maternal depression is of great interest.

One such alternative treatment may be maintaining a regular exercise routine throughout pregnancy and the postpartum period, which has been shown to both prevent and help treat depression symptoms among women able to safely engage in moderate to vigorous physical activity (11–15). Regular exercise has been linked with several beneficial effects, including improved body satisfaction (16), reduced physical discomfort (17), feelings of physical control despite somatic changes linked with pregnancy (18,19), and improved mood through the release of endorphins and neurotransmitters (18,20). It has therefore been recommended by the American College of Obstetricians and Gynecologists that pregnant women without medical contraindications engage in at least 150 minutes of moderate-intensity aerobic activity every week, divided into 30 minutes intervals most days of the week (21). Yet, many pregnant women do not exercise at the recommended levels, due in large part to fatigue, time constraints (e.g., work and childcare), and pregnancy-related physical limitations (e.g., back pain, swelling, and joint pain) (22,23). Differences are also apparent by location, with pregnant women living in urban areas exhibiting significantly higher levels of moderate physical activity compared to those living in rural areas, likely because of increased access to fitness centers (24,25).

Regardless of location, the benefits of exercise during pregnancy appears to be especially relevant during the COVID-19 pandemic, with women who reported at least 150 min of moderate intensity exercise exhibiting significantly lower scores for both anxiety and depression than those who did not (7). Yet the direct effects of the pandemic on exercise regimens among pregnant women remains poorly tested. Given the effects of shelter in place orders, it seems likely that many women's exercise routines will be disrupted by the pandemic, especially in metro areas (e.g., due to fitness centers and parks closing or a fear of viral exposure while exercising outside in a densely populated area). Conversely, women in less densely populated areas may feel safer going outside to engage in physical activities. However, the relationship between pandemic-related changes in exercise routines and depression risk has yet to be directly tested, and it is unclear how geographic location may impact this association.

We therefore use data drawn from the COVID-19 and Reproductive Effects (CARE) study to test the following two hypotheses among pregnant women living in the U.S.:

1) Participants who report changes in their exercise routines during the COVID-19 pandemic will demonstrate significantly higher depression scores compared to women who are able to maintain their exercise routine during the pandemic.
2) Compared to individuals who live in non-metro areas, participants living in metro areas (of all sizes) will be significantly more likely to report COVID-19-related changes in their exercise routines.

Materials and methods

Study design

The COVID-19 And Reproductive Effects (CARE) study was posted on social media platforms (Facebook, Twitter) and distributed via email to contacts working in maternity care and public health. Pregnant women over the age of 18 and living in the U.S. were invited to participate in a short survey assessing how the COVID-19 pandemic was impacting their medical care and birth plans. The data presented here were collected between April 16 – June 16, 2020. This study received ethical approval from Dartmouth College (STUDY00032045).

The survey was administered in REDCap, which automatically captures survey responses. Only women who met the inclusion criteria (i.e., living in the U.S., over 18 years of age, and currently pregnant) were eligible to participate in the survey. The survey completion rate (i.e., the percentage of those who consented to take the survey and actually went through to the end of the questionnaire) was 80%. During the study period, there were 1,862 surveys collected that included responses for all study variables. Data on depression symptomatology, exercise routines, and geographic location were collected, along with various covariates known to influence exercise patterns and depression risk.

Depression scores: Depression symptoms were screened using the Edinburgh Postnatal Depression Survey (EPDS), resulting in a participant score ranging from 0 (minimum) to 30 (maximum) (26). Depression scores were analyzed continuously.

Exercise routine changes: Participants were asked “has your exercise routine changed at all since the COVID-19 pandemic began?” Participants responded yes or no. Additionally, participants were asked the number of days per week (on average) they engaged in moderate exercise for at least 30 minutes.

Geographic location: Participants self-reported their zip codes. This information was used to generate a Rural-Urban Continuum Code (RUCC) for each respondent (27). The RUCC is based on the county located at the zip code center point, with each participant receiving a code on a 1-9 scale. For this analysis, the codes were collapsed as follows: 1) metro county of 1 million people or more, 2) metro county of 250,000 to 1 million people, 3) metro county of less than 250,000 people, and 4) all non-metro

counties. In addition, participants reported whether or not they lived in an area where they were currently required to shelter in place (i.e., not leave home, except for essential activities).

Age: Participants self-reported their age in years.

Current gestational week: Participants indicated their current gestational week.

Race/ethnicity: Race/ethnicity were self-reported and measured according to the Office of Management and Budget Standards (28). Native Hawaiian/Pacific Islander participants were re-classified as "Other" due to a small sample size (n = 3).

Household income: Participants indicated their household income from the following options: Less than $10,000 (1); $10,000 – $19,999 (2); $20,000 – $34,999 (3); $35,000 – $49,999 (4); $50,000 – $74,999 (5); $75,000 – $99,999 (6); $100,000+ (7). A composite household income variable was created for analysis: < $49,999, $50,000 – $99,999, and $100,000+.

Education: Participants selected their highest completed education from the following options: Some high school, no diploma (1); High school graduate, diploma or the equivalent (for example: GED) (2); Some college credit, no degree (3); Trade/technical/vocational training (4); Associate degree (5); Bachelor's degree (6); Master's degree (7); Professional degree (8); Doctorate degree (9). A composite education variable was created for analysis: less than a bachelor's degree, a bachelor's degree, or a degree beyond a bachelor's degree.

Financial stress: Participants were asked to rate whether they agreed or disagreed (Likert question; 5 options ranging from strongly disagree to strongly agree) with the statement "I am worried about my financial situation due to the COVID-19 crisis." Participants who stated that they agreed or strongly agreed with the statement were classified as having experienced COVID-19-related financial stress.

Risk status: Women were asked whether their pregnancy was classified as "high-risk." In addition, participants aged 35 or older were classified as "high-risk".

Statistical analysis

Data analyses were conducted using Stata 14. All continuous variables exhibited normal distributions, with skewness values within approximately ±0.5 and kurtosis values within approximately ±3. Multicollinearity was not detected between any variables; all VIF values were in an acceptable range of 1.01-1.65. Study descriptive statistics were calculated, and regression analyses were conducted to test the study hypotheses. Results were considered statistically significant at a p-value of less than 0.05.

Hypothesis 1: Linear regression analyses assessed whether exercise routine change during the COVID-19 pandemic was significantly associated with participant depression score.

Hypothesis 2: Logistic regression analyses tested whether a participant RUCC classification of “metro” was linked with significantly higher odds of reporting an altered exercise routine during the pandemic compared to a classification of “non-metro”.

All regression models adjusted for maternal age, education, income, financial stress, week of pregnancy at time of survey, race/ethnicity, and “high-risk” pregnancy.

Results

Sample characteristics and descriptive statistics

The study sample was spread out across all 50 states, including one participant in the U.S. territory of Puerto Rico (**Figure 1**). A clear majority of the sample (92.3%) indicated they lived in a location that required individuals to shelter in place (i.e., stay home, except for essential activities) at the time they completed the survey. Most women in the sample were physically active, with 56.4% of respondents reporting they engaged in moderate exercise for at least 30 minutes three or more days per week (versus the 17.9% of women who reported they were inactive).

Sample descriptive statistics are presented in **Table 1**. Mean participant age was 31 years old and the mean number of weeks pregnant was 26. Most respondents were white (87% of the sample), educated (77% had at least a bachelor's degree), lived in a metro area of 1 million or more people (61% of the sample), and were relatively high-income (55% reported an annual household income of $100,000 or more). Still, approximately 43% of participants reported that they worried about their financial situation due to the COVID-19 pandemic. Most participants (65%) did not indicate a high-risk pregnancy. Additionally, the majority of participants (60%) reported that their exercise routine had changed during the COVID-19 pandemic. Finally, participants varied in depression scores as measured by the EPDS scale, ranging from the minimum score of 0 all the way to the maximum score of 30; the mean EPDS score was 10.6.

Figure 1. Study participant locations across the United States. The number of participants in each zip code is summarized across United States counties and displayed as percentage of county population (29)**, with darker colors representing areas where participants make up a higher percentage of the county population (see legend). Data are presented for the sample used in the present analyses (n = 1,862). State and county spatial data courtesy Census.gov.**

Table 1. Descriptive statistics of model variables. Sample means (with standard deviation and range) or frequency (percent) of model variables, for 1,862 participants included in the analyses.

**Variable**	**Mean (SD; range)**
Age (years)	31.3 (4.30; 18-47)
Weeks pregnant at time of survey	26.0 (8.73; 0-41)
Edinburgh Postnatal Depression Survey (EPDS) score	10.6 (5.34; 0-30)
	**Frequency (%)**
Race/ethnicity:	
White	1,618 (86.9%)
Hispanic, Latino, or Spanish origin	112 (6.02%)
Black or African American	24 (1.29%)
Asian	60 (3.22%)
American Indian or Alaskan Native	12 (0.64%)

Other	36 (1.93%)
Household income:	
< $49,999	222 (11.9%)
$50,000 - $99,999	610 (32.8%)
$100,000+	1,030 (55.3%)
Education level:	
Less than a bachelor's degree	427 (22.9%)
Bachelor's degree	647 (34.8%)
Degree beyond a bachelor's degree	788 (42.3%)
Financial stress:	
Not worried	1,067 (57.3%)
Worried	795 (42.7%)
High-risk pregnancy:	
No	1,212 (65.1%)
Yes	650 (34.9%)
Exercise routine changed:	
No	736 (39.5%)
Yes	1,126 (60.5%)
RUCC code:	
Metro, 1 million+	1,133 (60.8%)
Metro, 250,000 – 999,999	394 (21.2%)
Metro, < 250,000	140 (7.52%)
Non-metro	195 (10.5%)

Hypothesis 1

Linear regression analyses were carried out to assess whether exercise routine change during the pandemic was significantly associated with depression score, measured using the EPDS (**Table 2**).

Participants who were older (B = -0.071, 95%CI: -0.140-(-0.001), p = 0.046), reported a higher household income (compared to < $49,999) of $50,000 - $99,999 (B = -0.944, 95%CI: -1.77-(-0.112), p = 0.026) or $100,000+ (B = -1.34, 95%CI: -2.22-(-0.470), p = 0.003), and were more highly educated (compared to less than a bachelor's degree) with either a bachelor's degree (B = -0.873, 95%CI: -1.56-(-0.189), p = 0.012) or a degree beyond a bachelor's degree (B = -1.13, 95%CI: -1.83-(-0.426), p = 0.002) exhibited significantly lower depression scores.

Conversely, participants who were Hispanic, Latino, or Spanish in origin (B = 1.29, 95%CI: 0.306-2.27, p = 0.010) or who reported experiencing COVID-19-related financial stress (B = 2.26, 95%CI: 1.78-2.75, p < 0.001) displayed significantly higher depression scores. Finally, as hypothesized, women who reported their exercise routine had changed during the pandemic demonstrated significantly higher depression scores (B = 0.917, 95%CI: 0.434-1.40, p < 0.001).

Table 2. Linear regression model assessing associations between reported exercise changes during the COVID-19 pandemic and depression, as measured by a continuous Edinburgh Postnatal Depression Survey (EPDS) score. Beta coefficients are provided with standard errors, 95% confidence intervals, and p-values for each variable included in the model.

Variable	B coefficient (SE, 95% CI)	p-value
Intercept	**12.45 (1.07, 10.36-14.5)**	**<0.001**
Age (years)	**-0.071 (0.035, -0.140-(-0.001))**	**0.046**
Weeks pregnant at time of survey	0.019 (0.014, -0.007-0.046)	0.156
Race/ethnicity:		
White	reference	
Hispanic, Latino, or Spanish origin	**1.29 (0.501, 0.306-2.27)**	**0.010**
Black or African American	0.039 (1.05, -2.02-2.10)	0.970
Asian	-1.01 (0.673, -2.33-0.311)	0.134
American Indian or Alaskan Native	-0.350 (1.48, -3.25-2.55)	0.813
Other	-0.456 (0.859, -2.14-1.23)	0.596

Household income:		
< $49,999	reference	
$50,000 - $99,999	**-0.944 (0.424, -1.77-(-0.112))**	**0.026**
$100,000+	**-1.34 (0.446, -2.22-(-0.470))**	**0.003**
Education level:		
Less than a bachelor's degree	reference	
Bachelor's degree	**-0.873 (0.349, -1.56-(-0.189))**	**0.012**
Degree beyond a bachelor's degree	**-1.13 (0.359, -1.83-(-0.426))**	**0.002**
Financial stress (no vs. yes)	**2.26 (0.246, 1.78-2.75)**	**<0.001**
High-risk pregnancy (no vs. yes)	0.561 (0.291, -0.009-1.13)	0.054
Exercise routine changed (no vs. yes)	**0.917 (0.246, 0.434-1.40)**	**<0.001**

Hypothesis 2

Logistic regression analyses were carried out to assess whether participant geographic location (i.e., non-metro vs. metro), as assessed by RUCC, was associated with significantly higher odds of reporting an altered exercise routine during the COVID-19 pandemic (**Table 3**). Participants who were farther along in their pregnancy had significantly lower odds of reporting changes to their exercise (OR = 0.986, 95%CI: 0.975-0.997, p = 0.010). Meanwhile, participants with a higher household income (compared to < $49,999) of $50,000 - $99,999 (OR = 1.56, 95%CI: 1.11-2.18, p = 0.010) or $100,000+ (OR = 1.58, 95%CI: 1.11-2.26, p = 0.012) and were more highly educated (compared to less than a bachelor's degree) with either a bachelor's degree (OR = 1.54, 95%CI: 1.17-2.03, p = 0.002) or a degree beyond a bachelor's degree (OR = 2.04, 95%CI: 1.53-2.71, p < 0.001) exhibited significantly higher odds of reporting exercise routine changes. Participants who reported experiencing pandemic-related financial stress (OR = 1.26, 95%CI: 1.03-1.54, p = 0.024) also displayed significantly higher odds of reporting exercise routine changes.

Finally, compared to participants living in a non-metro area, those living in a metro area of 1 million or more people (1.96, 95%CI: 1.42-2.71, p < 0.001), a metro area of 250,000 – 999,999 people (OR = 1.72, 95%CI: 1.21-2.46, p = 0.003), or a metro area of < 250,000 people (OR = 2.03, 95%CI: 1.29-3.20, p = 0.002) demonstrated significantly higher odds of reporting exercise routine changes, as hypothesized (**Figure 2**).

Table 3. Logistic regression model assessing associations between participant Rural-Urban Continuum Code (RUCC) and reported exercise change. Odds ratios are provided with standard errors, 95% confidence intervals, and p-values for each variable included in the model.

Variable	OR (SE, 95% CI)	p-value
Intercept	0.429 (0.196, 0.176-1.05)	0.063
Age (years)	1.00 (0.015, 0.974-1.03)	0.874
Weeks pregnant at time of survey	**0.986 (0.006, 0.975-0.997)**	**0.010**
Race/ethnicity:		
White	reference	
Hispanic, Latino, or Spanish origin	1.39 (0.295, 0.913-2.10)	0.125
Black or African American	1.04 (0.462, 0.433-2.48)	0.936
Asian	1.52 (0.467, 0.833-2.78)	0.173
American Indian or Alaskan Native	1.27 (0.772, 0.388-4.18)	0.691
Other	0.935 (0.329, 0.469-1.86)	0.848
Household income:		
< $49,999	reference	
$50,000 - $99,999	**1.56 (0.267, 1.11-2.18)**	**0.010**
$100,000+	**1.58 (0.289, 1.11-2.26)**	**0.012**
Education level:		
Less than a bachelor's degree	reference	
Bachelor's degree	**1.54 (0.216, 1.17-2.03)**	**0.002**
Degree beyond a bachelor's degree	**2.04 (0.297, 1.53-2.71)**	**<0.001**

Financial stress (no vs. yes)	**1.26 (0.129, 1.03-1.54)**	**0.024**
High-risk pregnancy (no vs. yes)	1.10 (0.133, 0.870-1.40)	0.420
RUCC:		
Non-metro	reference	
Metro, 1 million+	**1.96 (0.323, 1.42-2.71)**	**<0.001**
Metro, 250,000 – 999,999	**1.72 (0.313, 1.21-2.46)**	**0.003**
Metro, < 250,000	**2.03 (0.470, 1.29-3.20)**	**0.002**

Figure 2. Percentage of study participants reporting change in exercise routine by United States county for (A) metro counties, and (B) non-metro counties. Map colors represent the proportion of participants in the sample reporting exercise routine changes during the pandemic within a given county (see legend), ranging from blue (a low percentage of sample participants reported exercise changes) to red (a high percentage of sample participants reported exercise routine changes). Metro counties are displayed in gray-scale according to the USDA Rural-Urban Continuum Codes (RUCC), while non-metro counties are displayed in white (27)**. State and county spatial data courtesy Census.gov.**

Discussion

The study findings support both hypotheses. Women who reported exercise changes during the COVID-19 pandemic exhibited significantly higher depression scores compared to those who reported no changes. Moreover, individuals living in metro areas of all sizes were significantly more likely to report exercise changes compared to women living in non-metro areas, potentially because women living in more rural areas felt safer venturing outside for walks or other forms of physical activity given the lower population density and less perceived risk of viral exposure. Although cross-sectional and preliminary in nature, these results align with existing studies demonstrating the beneficial effects of exercise during pregnancy (11–15), suggesting moderate exercise may serve as a tool for supporting mental health during pregnancy.

Further, the association documented here suggests one possible mechanism by which the ongoing COVID-19 pandemic may impair maternal mental health. Business closures and stay at home orders resulting from the pandemic may directly inhibit one's ability to engage in physical activity. The majority of study participants (92.3%) indicated they were currently experiencing shelter in place orders, likely leading to disruptions in daily routines, including ability to engage in normal exercise activities. This idea is generally supported by preliminary analyses from the sample suggesting that a high percentage of participants (47.4%) explicitly reported exercising less following the onset of the pandemic, while many fewer respondents reported they were exercising more (9.12%). This difference suggests that exercise changes during the pandemic have generally resulted in less physical activity. This may be linked with gym, recreation center, and park closures that disrupt normal exercise routines, especially in urban areas where there is limited green space available for outdoor physical activity.

The disruption of exercise routines may have several negative effects contributing to elevated depression symptomatology, including decreased body satisfaction, increased physical discomfort, perceptions of not being in control (of both physical changes associated with pregnancy and everyday activities), as well as poor mood (16–20). Thus, maternal depression risk—already a concern among pregnant women prior to the COVID-19 pandemic—may be exacerbated during the pandemic partly because of disruptions to daily routines, including typical exercise regimens. The effects of altered exercise routines on maternal health may be especially relevant in the CARE study sample. These data were collected from women living in the U.S., the country hardest hit by the COVID-19 pandemic thus far (1). The prolonged nature of the lockdowns and current uptick of infections in many parts of the country will likely continue to disrupt normal routines, including ability to exercise, for the foreseeable future. Consequently, the association between depression score and changes in exercise routine documented here suggest that maternal depression screenings should account for the impact of COVID-19 on physical activity levels, in addition to other effects of the pandemic on maternal well-being.

Additionally, providers should consider how ability to engage in physical activity during the pandemic may influence maternal mental health, particularly among women living in urban areas. Providers and/or fitness specialists could work with pregnant women and new mothers to find simple routines (e.g., exercises that can be completed safely from home without specialized equipment and which do not require much open space) to encourage physical activity during the pandemic across all demographics and living conditions. Indeed, it has been suggested that household chores, walks, gardening, and online fitness classes may be possible alternatives to support maternal physical and mental health (7).

However, efforts must be made to recommend exercises tailored to each individual's living conditions and physical health, as the negative effects of changed exercise routines are not likely to be experienced equally across all sociodemographic groups. Specifically, higher-income women with access to more resources may find it easier to adopt alternative forms of exercise as the pandemic continues (e.g., purchase home gym equipment or exercise videos), allowing them stay physically active even if they live in urban areas where they cannot easily access a gym or exercise outside. Conversely, lower-income communities located in densely populated areas without infrastructure (e.g., green space and sidewalks) to support safe outdoor activities during COVID-19 may be more substantially impacted by pandemic-related public facility closures and shelter in place orders. However, this remains to be directly tested using a more diverse sample.

Limitations

It should be noted that despite the strengths of these analyses (e.g., large sample size and participants from across the country), several important study limitations exist. First, this study is cross-sectional, making it difficult to establish causality. For instance, it is not possible to definitively establish

whether the significant relationship observed between exercise routine change during the pandemic and depression score is due to disrupted exercise routines leading to later depression or to maternal depression leading to decreased motivation to exercise. The relationship between physical activity and depression appears to be bidirectional in nature, with exercise protecting against depression but baseline depression also subsequently decreasing physical activity levels (30). Longitudinal data collection is needed to establish these causal relationships.

In addition, due to the use of convenience sampling, these data are not representative of the U.S. population as a whole; white, educated, wealthy women are overrepresented in the present sample (31). Additional work is needed to determine whether the associations observed here are also evident across a more representative, diverse sample of the U.S. population. Finally, quantitative data on pre-pandemic exercise patterns were not collected, making it difficult to establish whether the patterns documented here partly reflect pre-existing physical activity differences. For example, previous work indicates that pregnant women living in urban areas exhibit higher levels of moderate exercise compared to those living in rural areas (24,25); these women may therefore be less likely to report exercise changes as they were not regularly exercising prior to the pandemic. Still, this pattern does not detract from the finding that urban women appear to be exercising less during the pandemic, which may negatively impact their physical and mental well-being.

Conclusions

The COVID-19 pandemic has far-ranging effects on both mental and physical health. Certain segments of the population may be especially impacted by the pandemic; including pregnant women, who already suffer from an elevated depression risk compared to the general public (8). The findings presented here support previous work and suggest an association exists between ability to maintain an

exercise routine during pregnancy and depression risk. Providers should explicitly ask patients how the pandemic has impacted their exercise routines and consider this a risk factor for depression. Moreover, efforts should be made to recommend exercises that are tailored to individual living conditions (e.g., whether they can safely exercise outdoors) and physical health.

Specifically, the findings presented here indicate that women living in metro areas were significantly more likely to report exercise routine changes during the pandemic than women living in non-metro areas. This may signify an opportunity for interventions—especially in urban areas—which rely on simple exercises that can safely be performed at home without specialized equipment (e.g., squats, lunges, side-lying leg lifts, etc.). Importantly, exercise recommendations may be most effective if communicated by providers who understand the personal health and space limitations of each individual patient. Exercise represents a potential non-pharmaceutical tool to support both mental and physical health among pregnant women, both during and after the pandemic.

Acknowledgements

We would like to thank Nadia Clement for her assistance building the online survey and designing the recruitment flyers. We would also like to thank Margaret Sherin, Gloriuese Uwizeye, and Chlöe Sweetman for their valuable feedback on the questionnaire during development. Further, we express our gratitude to Grace Alston, Cecily Craighead, Amanda Lu, and Rebecca Milner for their assistance in data coding. Finally, we thank Matthew Nagy for his assistance in data cleaning.

References

1. Center for Systems Science and Engineering (CSSE). COVID-19 Dashboard [Internet]. John Hopkins University; 2020 [cited 2020 Jul 30]. Available from: https://coronavirus.jhu.edu/map.html

2. Dashraath P, Wong JLJ, Lim MXK, Lim LM, Li S, Biswas A, et al. Coronavirus disease 2019 (COVID-19) pandemic and pregnancy. American Journal of Obstetrics and Gynecology. 2020 Jun 1;222(6):521–31.

3. Abbas AM, Ahmed OA, Shaltout AS. COVID-19 and maternal pre-eclampsia: A synopsis. Scandinavian Journal of Immunology. n/a(n/a):e12918.

4. Narang K, Enninga EAL, Gunaratne MDSK, Ibirogba ER, Trad ATA, Elrefaei A, et al. SARS-CoV-2 Infection and COVID-19 During Pregnancy: A Multidisciplinary Review. Mayo Clinic Proceedings. 2020 May;S0025619620305164.

5. Shanes ED, Mithal LB, Otero S, Azad HA, Miller ES, Goldstein JA. Placental Pathology in COVID-19. Am J Clin Pathol. 2020 Jun 8;154(1):23–32.

6. Centers for Disease Control and Prevention (CDC). Coronavirus Disease 2019: People with Certain Medical Conditions. [Internet]. 2020 [cited 2020 Jul 3]. Available from: https://www.cdc.gov/coronavirus/2019-ncov/need-extra-precautions/people-with-medical-conditions.html#pregnancy.

7. Davenport MH, Meyer S, Meah VL, Strynadka MC, Khurana R. Moms Are Not OK: COVID-19 and Maternal Mental Health. Front Glob Womens Health [Internet]. 2020 [cited 2020 Jun 23];1. Available from: https://www.frontiersin.org/articles/10.3389/fgwh.2020.00001/full

8. Centers for Disease Control and Prevention (CDC). Reproductive Health: Depression During and After Pregnancy [Internet]. 2020 [cited 2020 Jul 3]. Available from: https://www.cdc.gov/reproductivehealth/features/maternal-depression/index.html

9. Thayer ZM, Gildner TE. COVID-19-associated financial stress predicts significantly higher depression symptoms among pregnant American women. American Journal of Human Biology. In Review;

10. Sherin M, Gildner TE, Thayer ZM. The Association between COVID-19-Related Changes to Pregnant Women's Work-Plans and Prenatal Depression. Obstetrics & Gynecology. In Review;

11. El-Rafie MM, Khafagy GM, Gamal MG. Effect of aerobic exercise during pregnancy on antenatal depression. Int J Womens Health. 2016 Feb 24;8:53–7.

12. Poyatos-León R, García-Hermoso A, Sanabria-Martínez G, Álvarez-Bueno C, Cavero-Redondo I, Martínez-Vizcaíno V. Effects of exercise-based interventions on postpartum depression: A meta-analysis of randomized controlled trials. Birth. 2017;44(3):200–8.

13. Prather H, Spitznagle T, Hunt D. Benefits of Exercise During Pregnancy. PM&R. 2012 Nov;4(11):845–50.

14. Robledo-Colonia AF, Sandoval-Restrepo N, Mosquera-Valderrama YF, Escobar-Hurtado C, Ramírez-Vélez R. Aerobic exercise training during pregnancy reduces depressive symptoms in nulliparous women: a randomised trial. Journal of Physiotherapy. 2012 Mar 1;58(1):9–15.

15. Vargas-Terrones M, Barakat R, Santacruz B, Fernandez-Buhigas I, Mottola MF. Physical exercise programme during pregnancy decreases perinatal depression risk: a randomised controlled trial. Br J Sports Med. 2019 Mar;53(6):348–53.

16. Boscaglia N, Skouteris H, Wertheim EH. Changes in body image satisfaction during pregnancy: A comparison of high exercising and low exercising women. Australian and New Zealand Journal of Obstetrics and Gynaecology. 2003;43(1):41–5.

17. Nascimento SL, Surita FG, Cecatti JG. Physical exercise during pregnancy: a systematic review. Current Opinion in Obstetrics and Gynecology. 2012 Dec;24(6):387–394.

18. Mandolesi L, Polverino A, Montuori S, Foti F, Ferraioli G, Sorrentino P, et al. Effects of Physical Exercise on Cognitive Functioning and Wellbeing: Biological and Psychological Benefits. Front Psychol [Internet]. 2018 [cited 2020 Jun 25];9. Available from: https://www.frontiersin.org/articles/10.3389/fpsyg.2018.00509/full

19. Weinberg RS, Gould D. Foundations of sport and exercise psychology. Human Kinetics; 2014.

20. Mikkelsen K, Stojanovska L, Polenakovic M, Bosevski M, Apostolopoulos V. Exercise and mental health. Maturitas. 2017 Dec 1;106:48–56.

21. American College of Obstetricians and Gynecologists (ACOG)). Exercise During Pregnancy [Internet]. 2019 [cited 2020 Jul 16]. Available from: https://www.acog.org/patient-resources/faqs/pregnancy/exercise-during-pregnancy

22. Cramp AG, Bray SR. A Prospective Examination of Exercise and Barrier Self-efficacy to Engage in Leisure-Time Physical Activity During Pregnancy. Ann Behav Med. 2009 Jun 1;37(3):325–34.

23. Fell DB, Joseph KS, Armson BA, Dodds L. The Impact of Pregnancy on Physical Activity Level. Matern Child Health J. 2009 Sep;13(5):597–603.

24. Marshall ES, Bland H, Melton B. Perceived Barriers to Physical Activity Among Pregnant Women Living in a Rural Community. Public Health Nursing. 2013;30(4):361–9.

25. Melton B, Marshall E, Bland H, Schmidt M, Guion WK. American rural women's exercise self-efficacy and awareness of exercise benefits and safety during pregnancy. Nursing & Health Sciences. 2013;15(4):468–73.

26. Cox J, Holden J, Sagovsky R. Edinburgh postnatal depression scale (EPDS). Br J psychiatry. 1987;150:782–6.

27. United States Department of Agriculture (USDA). Rural-Urban Continuum Codes [Internet]. 2013 [cited 2020 Jul 2]. Available from: https://www.ers.usda.gov/data-products/rural-urban-continuum-codes.aspx

28. Office of Management and Budget Standards. Revisions to the Standards for the Classification of Federal Data on Race and Ethnicity [Internet]. 1997. Available from: https://www.govinfo.gov/content/pkg/FR-1997-10-30/pdf/97-28653.pdf

29. United States Census Bureau. TIGER/Line Shapefile, 2017, 2010 nation, U.S., 2010 Census Urban Area National [Internet]. 2019. Available from: https://catalog.data.gov/dataset/tiger-line-shapefile-2017-2010-nation-u-s-2010-census-urban-area-national

30. Roshanaei-Moghaddam B, Katon WJ, Russo J. The longitudinal effects of depression on physical activity. General Hospital Psychiatry. 2009 Jul 1;31(4):306–15.

31. Martin JA, Hamilton BE, Osterman MJ, Driscoll AK. Births: Final data for 2018. National vital statistics reports [Internet]. 2019;68(13). Available from: https://stacks.cdc.gov/view/cdc/82909

Supporting information

S1 Checklist. STROBE Checklist.

Figure

Click here to access/download;Figure;Figure 1.tiff

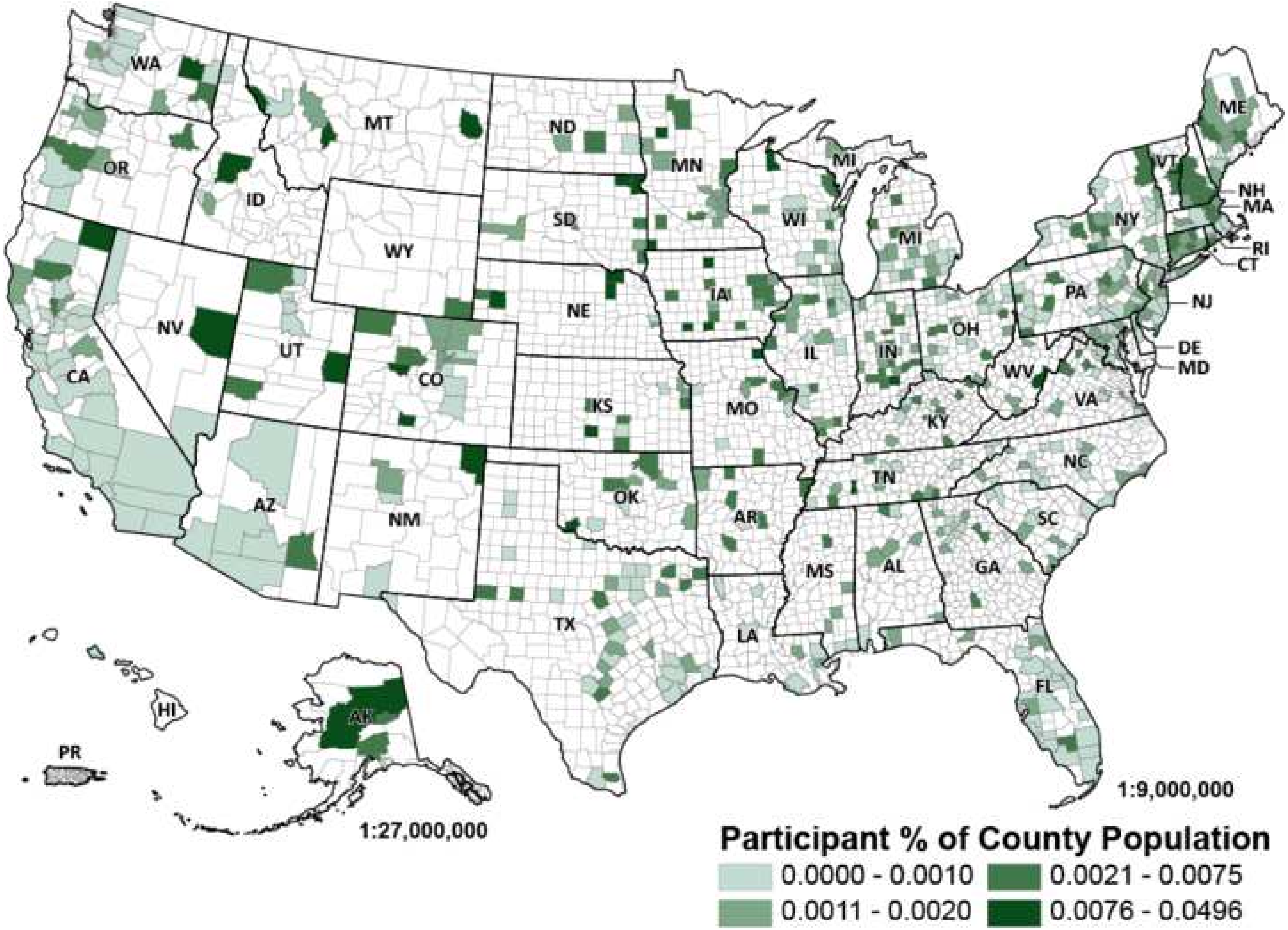

Click here to access/download;Figure;Figure 2.tiff

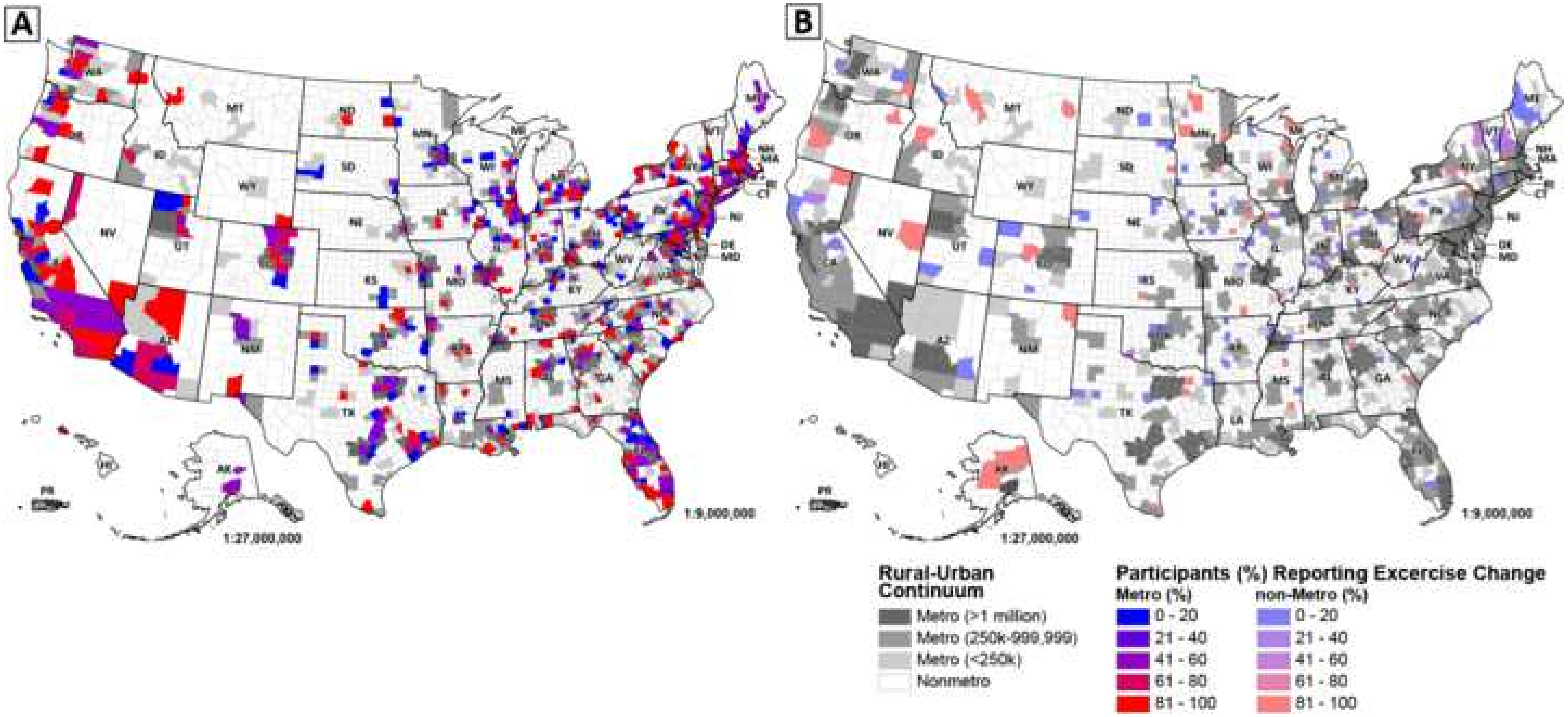

Click here to access/download

Supporting Information

Gildner

STROBE_checklist_v4_combined_PlosMedicine.docx
