# Supplementary material for: Exercise routine change is associated with prenatal depression scores during the COVID-19 pandemic among pregnant women across the United States
Source: PLoS One. 2020 Dec 21;15(12):e0243188. doi: 10.1371/journal.pone.0243188 (PMC7751871; doi:10.1371/journal.pone.0243188)
Supplement: Attachment — Submitted filename: Review comments.docx [file pone.0243188.s002.docx]

Dear Sir/Mam

Please find bellow the requested review regarding manuscript entitled: Exercise routine change is associated with prenatal depression scores during the COVID-19 pandemic among pregnant women across the United States. The article contains a lot of useful information on the issue. The topic is very interesting and use of sources is appropriate. Some revisions are necessary: The aim of the study is clear, the title is informative and relevant, but the abstract section involve too much information. The authors should not write such details about their manuscript. As regards the references, are relevant, but not so recent, referenced correctly, and there are appropriate key studies included. However, the authors should add some more, in order to establish their findings.

Introduction/ background

It is quite clear what is already known about this topic and the research question is clearly outlined. The research question is not justified clearly, given what is already known about the topic. Some chapters are lengthy related to others. The authors should keep uniformity about the length of the chapters.

Overall

The article contains a lot of useful information and the topic is very interesting. Some revisions are necessary: The author should provide more information in some chapters and omit some others. In addition, they should conclude a chapter with the limitations of the studies mentioned.

Positive: There are some strengths of the article that could have an impact in the field, such as the topic and its impact on health care.
